# Parenting Across European Cultures: Parental Practices and Adolescent Adjustment in Germany and Spain

**DOI:** 10.3390/bs16050638

**Published:** 2026-04-24

**Authors:** Joan García-Perales, Joan García-Ruiz, Desamparados Ruiz Gil, Margarete Imhof

**Affiliations:** 1 Department of Methodology of the Behavioral Sciences, University of Valencia, Av. Blasco Ibáñez, 21, 46010 Valencia, Spain; 2 Institute of Chemical Technology (ITQ), Valencia Polytechnic University (UPV), Camí de Vera, s/n, Algirós, 46022 Valencia, Spain; joagarru@itq.upv.es; 3 High School Chemistry Department, IES Marxadella, Conselleria d’Educació, C. Pare Mendez, 151, 46900 Torrent, Spain; d.ruizgil@edu.gva.es; 4 Department of Educational Psychology, Johannes Gutenberg-University of Mainz, Binger Strasse 14–16, 55099 Mainz, Germany; imhof@uni-mainz.de

**Keywords:** cultural context, parenting styles, warmth and strictness, adolescent adjustment, German and Spanish samples

## Abstract

This study examines whether the association between parenting styles and adolescent adjustment reflects universal principles or culturally embedded processes, comparing adolescents from Germany (*n* = 395) and Spain (*n* = 331). Grounded in the bidimensional model of parental socialization (warmth × strictness), four styles were identified: authoritative, indulgent, authoritarian, and neglectful. Participants (Mage = 15.6 years) completed measures of parental socialization (*ESPA29*) and multidimensional self-concept (*AF5*); academic achievement was obtained from school records; and substance use was self-reported. A cross-sectional design was employed. Multivariate analyses of variance that revealed warmth was positively associated with all self-concept domains and negatively with substance use, whereas strictness showed weak or negative links. Significant Parenting Style × Country interactions emerged for academic self-concept, achievement, and substance use. In the Spanish sample, indulgent parenting exhibited a distinct pattern, particularly with respect to academic self-concept. Among German adolescents, both indulgent and authoritative styles yielded favorable outcomes, with authoritative parenting demonstrating protective effects against substance use. These findings suggest that the effectiveness of the authoritative style may not be uniform across contexts and underscore the importance of cultural factors in defining optimal parenting, supporting a contextualist model of adolescent socialization across European contexts.

## 1. Introduction

The study of parenting across cultures is central to developmental psychology, as it allows an examination of whether associations between family socialization and adolescent adjustment reflect universal processes or societal specific patterns (Lansford, 2022; Bornstein & Putnick, 2016; Pinquart & Gerke, 2019). European cross-cultural research provides an appropriate framework for this question, given the variability in family norms, parenting practices, and educational expectations (Selin, 2014; Lee et al., 2023). In this context, comparisons between Germany (Ostner & Stolberg, 2015; Rönsch, 2020) and Spain (F. García & Gracia, 2009, 2014; F. García et al., 2019; O. F. García et al., 2020) are particularly informative, as both countries differ in family cohesion and normative beliefs about parental roles, which may shape the developmental meaning of parenting dimensions such as warmth and strictness (Rohner & Lansford, 2017; O. F. García et al., 2020). The present study examines these associations by comparing parenting practices, dimensions, and styles, and their links with adolescent adjustment in German and Spanish samples.

### 1.1. Parental Socialization and the Bidimensional Model

Parental socialization is a predominantly parent-driven but bidirectional developmental process through which children and adolescents internalize the cultural norms, values, and behavioral standards necessary for the effective adaptation to their social environment. This process culminates in the development of autonomous and socially responsible adults (Baumrind, 1978; Pinquart & Gerke, 2019). During adolescence—a particularly sensitive developmental period—core dimensions of psychosocial adjustment consolidate, including self-concept, academic achievement, and engagement in risk behaviors (Fuentes et al., 2015; J. García-Perales, 2011; J. R. García-Perales & Martínez, 2012; J. R. García-Perales, 2013; Kerr & Stattin, 2000; Martínez & García, 2007). Despite adolescents’ increasing autonomy, family relationships remain a central developmental context, as parents continue to provide emotional support, behavioral regulation, and normative guidance (Alonso-Stuyck, 2019; Deater-Deckard et al., 2011; F. García et al., 2019; Steinberg, 2005).

**Figure 1:**
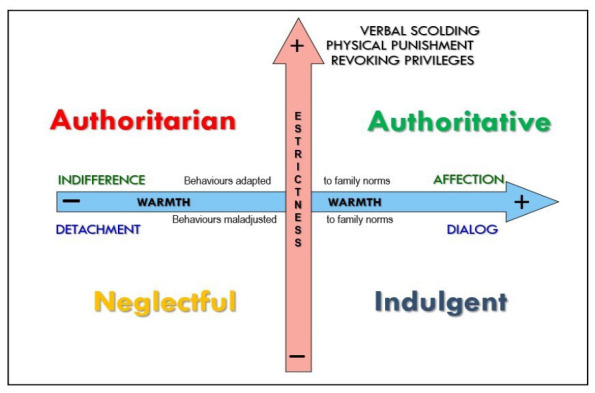
Bidimensional model of parental socialization (Musitu & García, 2001).

Research on parental socialization has traditionally conceptualized parenting styles along two orthogonal and theoretically independent dimensions: parental warmth and parental strictness (Baumrind, 1966, 1967; Darling & Steinberg, 1993; Martínez et al., 2017; Rohner & Lansford, 2017; Rohner & Smith, 2019; Smetana, 1995, 2017; Steinberg, 2005), although alternative terminologies with comparable meanings have been employed in the literature (Martínez et al., 2017, 2019). Based on the bidimensional model of parental socialization developed by Musitu and García (2001) (Figure 1), the seven parenting practices define two parental dimensions, and the intersection of these dimensions gives rise to the four parenting styles.

Warmth encompasses support, affection, open communication, and reasoning. High warmth is expressed through affection when adolescents comply with family norms and through dialogue when behavior deviates from expectations. Low warmth is reflected in indifference and detachment (F. García & Gracia, 2009, 2010; Maccoby & Martin, 1983). In contrast, strictness refers to demandingness, behavioral control, and firmness in the enforcement of family rules. It captures the extent to which parents implement regulatory strategies to correct norm-violating behavior, typically operationalized through practices such as verbal scolding, revoking privileges, and, in some cases, physical punishment.

The orthogonal combination of warmth and strictness yields four parenting styles: authoritative (high warmth/high strictness), indulgent (high warmth/low strictness), authoritarian (low warmth/high strictness), and neglectful (low warmth/low strictness) (Maccoby & Martin, 1983; Darling & Steinberg, 1993; Lamborn et al., 1991). This typology has demonstrated strong heuristic value in explaining the variability in child and adolescent adjustment.

Research on parenting requires a clear distinction between parenting styles, parenting practices, and parenting dimensions, alongside a careful consideration of their cultural interpretation. Parenting styles refer to relatively stable, higher-order patterns that characterize the emotional climate of the parent–child relationship, typically defined by the combination of warmth and control. Parenting practices denote specific, goal-directed behaviors used in concrete situations, such as monitoring or discipline. Parenting dimensions represent the underlying continuous constructs, including responsiveness, demandingness, and psychological control, which can be assessed independently and combined to derive broader styles. The meaning and developmental implications of these constructs are shaped by cultural norms and socialization goals, so behaviors interpreted as controlling in one context may be viewed as normative or supportive in another.

### 1.2. Parenting Across Cultural Contexts

Early research conducted primarily in Anglo-Saxon, European-American samples identified the authoritative style as the most effective parenting pattern for promoting psychosocial competence and academic success (Baumrind, 1971, 1972, 1991; Lavrič & Naterer, 2020; Steinberg et al., 1989, 1994). Adolescents raised in authoritative households consistently exhibited higher self-regulation, social competence, academic achievement, and lower levels of problem behavior (Lamborn et al., 1991; Lavrič & Naterer, 2020).

Accumulating cross-cultural evidence has increasingly questioned the presumed universality of traditional parenting style models. Meta-analytic studies show that both the strength and direction of associations between parenting styles and developmental outcomes vary substantially across cultural contexts (F. García et al., 2019; J. R. García-Perales & Martínez, 2012; Martínez et al., 2019; Pinquart, 2017; Pinquart & Kauser, 2018). This variability suggests that parenting practices acquire their developmental significance partly through their cultural normativeness, understood as the extent to which they are perceived as legitimate and customary within a given society (Lansford, 2022). In line with this perspective, research conducted with ethnic minority groups in the United States and with non-Western families indicates that authoritarian practices do not consistently predict maladjustment and may even be associated with adaptive outcomes when strict control is culturally interpreted as a form of protection and parental investment (Chao, 1994; Deater-Deckard & Dodge, 1997; Deater-Deckard et al., 2011; Steinberg et al., 1992; Torres-Villa, 1995; Wang & Phinney, 1998; Zayas & Solari, 1994). Similarly, studies in Southern European and Latin American contexts have reported that the indulgent style is linked to levels of psychosocial adjustment comparable to, or even exceeding, those associated with the authoritative style (F. García & Gracia, 2009, 2010; J. García-Perales et al., 2026; Martínez & García, 2007; Rodrigues et al., 2013).

In Mediterranean contexts, where family cohesion and emotional closeness hold strong normative value, parental warmth appears to play a more central role than behavioral strictness in promoting adolescent adjustment. Recent findings from Germany further support the relevance of warmth-based parenting (Ostner & Stolberg, 2015; Rönsch, 2020; Walper & Kreyenfeld, 2022). Cross-cultural and longitudinal studies grounded in Self-Determination Theory suggest that supportive parenting profiles characterized by warmth and autonomy support predict positive developmental trajectories, whereas controlling or low-support profiles are associated with poorer outcomes. Importantly, Germany’s internal cultural diversity and changing structural conditions (e.g., teleworking arrangements) further underscore the contextual embeddedness of parenting effects. Together, these findings question the universal prescription of the authoritative style and suggest that the developmental effectiveness of parenting styles must be interpreted within broader cultural and normative frameworks.

### 1.3. Parenting Styles, Gender, and Age

The effects of parenting are moderated by adolescents’ characteristics, particularly gender and age (Steinberg, 2001; Alonso-Stuyck et al., 2018). Adolescence involves progressive changes in autonomy that reshape family dynamics and perceptions of parental authority (Steinberg, 2005). Evidence indicates that parents may adopt differentiated practices depending on the child’s gender, often reporting higher levels of monitoring and communication with daughters than with sons (Deater-Deckard & Dodge, 1997; Pinquart, 2017). Age-related differences have also been documented, with parental warmth and control typically decreasing as adolescents grow older (Alonso-Stuyck et al., 2018; Jensen et al., 2024). These variations suggest that the impact of parenting styles should be considered within a developmental and sociodemographic context.

### 1.4. Parenting Styles and Adolescent Adjustment

Adolescent adjustment is commonly examined across three central domains: self-concept, academic achievement, and substance use. Self-concept represents a core indicator of internal well-being and is closely linked to behavioral, emotional, and social functioning (Harter, 2006). Meta-analytic evidence shows moderate positive associations between authoritative parenting and self-esteem, whereas authoritarian and neglectful styles are negatively associated with self-evaluations. Pinquart and Gerke (2019) concluded that authoritative parenting was positively associated with adolescents’ self-esteem and psychosocial adjustment, while authoritarian and neglectful styles showed negative associations across cultural contexts. Notably, research in Spain indicates that adolescents from indulgent families may report equal or even higher self-esteem and psychosocial adjustment than those from authoritative families, suggesting cultural moderation effects (Fuentes et al., 2015; F. García & Gracia, 2009; O. F. García et al., 2018; J. García-Perales et al., 2026; Martínez-Ferrer et al., 2019). For instance, Martínez et al. (2019) found that indulgent parenting was associated with equal or better self-esteem and internalization of values compared to authoritative parenting among Spanish adolescents. More recent studies support these findings across adolescence (Cuadri et al., 2025; F. García et al., 2019, O. F. García et al., 2020; Pérez-Gramaje et al., 2020; Sánchez-Urrea et al., 2024), reinforcing the relevance of parental warmth in Southern European contexts.

Academic achievement has been associated with parenting styles, although the effect sizes are generally small and moderated by contextual factors such as socioeconomic status and cultural norms. Early Anglo-Saxon research highlighted the benefits of authoritative parenting (Baumrind, 1967; Steinberg et al., 1994), but subsequent meta-analyses indicate modest positive associations, with authoritarian parenting showing null or negative links in Western contexts. Evidence from European samples further suggests that both indulgent and authoritative styles are related to more favorable academic outcomes than authoritarian and neglectful styles, underscoring the relevance of parental warmth across cultural settings (Fuentes et al., 2015; F. García et al., 2019; J. R. García-Perales & Martínez, 2012; Martínez-Ferrer et al., 2019; Pinquart & Kauser, 2018).

Substance use constitutes a major public health concern during adolescence (Farnia et al., 2024). While early studies identified authoritative parenting as the most protective style (Pinquart & Lauk, 2025; Steinberg, 2005; Steinberg et al., 1994), more recent European research demonstrates that indulgent and authoritative styles are similarly associated with lower levels of alcohol, tobacco, and illicit drug use. A longitudinal study published in *Adolescents* found that adolescents perceiving indulgent or authoritative parenting reported significantly lower substance use compared to those from neglectful families (Adalbjarnardottir & Hafsteinsson, 2001; Alati et al., 2014; Fuentes et al., 2015). Likewise, Calafat et al. (2014), O. F. García et al. (2020), and Palacios et al. (2022) reported that low parental warmth (authoritarian and neglectful styles) predicted poorer psychosocial trajectories, including greater behavioral risk. These findings suggest that parental warmth, rather than strictness per se, may constitute the primary protective mechanism in several European contexts, a conclusion also supported by cross-national reviews (Pinquart, 2017).

Overall, converging cross-sectional and longitudinal evidence indicates that parenting styles characterized by high warmth—authoritative and indulgent—are consistently associated with positive psychosocial adjustment indicators across domains, whereas authoritarian and neglectful styles are associated with distinct developmental patterns that may vary depending on contextual factors. Cultural context appears to moderate the relative benefits of strictness, particularly in Southern European countries, where indulgent parenting often performs as well as or higher than authoritative parenting in predicting adolescent well-being.

However, recent empirical research has continued to document less favorable outcomes associated with indulgent parenting, particularly in domains related to self-regulation, mental health, and behavioral adjustment. For instance, Grolnick (2015) and subsequent work have emphasized that low levels of parental structure and demandingness are associated with poorer self-regulatory capacities in children and adolescents. More recent evidence has linked permissive parenting to increased risk-taking behaviors and reduced behavioral control, highlighting the role of insufficient parental monitoring (Lansford et al., 2018). In addition, meta-analytic findings by Pinquart and Kauser (2018) and Feng and Cui (2023) indicate that indulgent parenting is associated with higher levels of externalizing problems and lower academic achievement compared to more structured parenting styles. Recent studies have also pointed to links between permissive parenting and poorer emotional regulation and psychological well-being, particularly when low demandingness limits the development of self-control (e.g., Almeida & Santos, 2024). Taken together, these findings suggest that, despite cultural variability, contemporary evidence continues to associate indulgent parenting with elevated developmental risk, especially in relation to behavioral regulation and psychosocial adjustment.

### 1.5. The Present Study

The present study aims to examine how parenting practices, dimensions, and styles are differentially associated with adolescent adjustment, comparing a Northern European sample (Germany) and a Southern European sample (Spain). Specifically, it investigates the relationship between parenting and adolescents’ self-concept, academic achievement, and substance use, while assessing whether these associations vary across distinct cultural and normative contexts.

Three hypotheses are proposed:

**H1.**  *Parenting dimensions and parenting styles are expected to be significantly associated with adolescents’ adjustment outcomes.*

**H2.**  *Exploratorily, parenting practices are expected to vary as a function of adolescents’ gender, age, and country of residence.*

**H3.**  *The magnitude and direction of the associations between parenting styles and adolescents’ adjustment outcomes are expected to differ across national contexts.*

By directly comparing a Spanish and a German sample, this study contributes to the ongoing debate on the cultural generalizability of parenting models by examining whether the effects of parenting styles on adolescents’ psychosocial adjustment are modulated by cultural context, thereby critically reassessing the presumed universal differential effectiveness of the authoritative style.

## 2. Materials and Methods

### 2.1. Participants

The sample consisted of 726 adolescents, enrolled in high schools located in large metropolitan areas along the eastern coast of Spain and in the central-western region of Germany, of which 418 were female (57.6%) and 308 male (42.4%). The mean age of participants was 15.8 years (*SD* = 1.70) for female and 15.4 years (*SD* = 1.60) for male. Regarding country-specific distributions, the Spanish subsample included 203 female (28.0%) and 118 male (17.6%), with a mean age of 15.9 years (*SD* = 1.95) for female and 15.4 years (*SD* = 1.70) for male. The German subsample consisted of 215 female (29.6%) and 180 male (24.8%), whose mean age was 15.8 years (*SD* = 1.40) for female and 15.4 years (*SD* = 1.50) for male.

### 2.2. Procedure

Data were gathered from 11 educational institutions (7 in Spain and 4 in Germany), which were selected through simple random sampling from a list of eligible schools (Steinberg et al., 1989; Martínez et al., 2011).

In both countries, we selected adolescents from middle-class neighborhoods who (a) lived in two-parent nuclear families, with a mother or primary female caregiver and father or primary male caregiver, and (b) their parents were born in the country of each sample (Spain and Germany) following criteria from other studies (F. García & Gracia, 2009).

The minimum sample size was estimated using an a priori power analysis G*Power version 3.1.9.7 (Faul et al., 2009). A medium–small effect size (f = 0.16) was assumed, estimated from ANOVAs by Lamborn et al. (1991) in a univariate F-test between the four parenting style groups (Pérez-Gramaje et al., 2020). A priori power analyses (α = 0.05, 1 − β = 0.95, and *f* = 0.16) showed a minimum sample size of 700 participants (Faul et al., 2009; Kang, 2021). All statistical analyses were performed with IBM SPSS statistics (Version 29) (IBM Corp., 2022).

The study was conducted in accordance with the ethical principles of research involving human subjects (Declaration of Helsinki). Ethical approval was obtained from the Ethics Committee under approval number (08/25). Participants did not receive financial compensation. All participants provided electronic informed consent, including parental authorization, and were informed of the voluntary nature of participation, the confidentiality and anonymity of the data, and their right to withdraw at any time. The necessary permissions were obtained from both the educational authorities and the parents. Questionnaires were administered online during regular school hours, with standardized instructions and in the presence of researchers. Completion time was approximately 25 min.

### 2.3. Instruments

#### 2.3.1. Parental Socialization

Parental socialization was assessed using the Parental Socialization Scale *ESPA29* (Musitu & García, 2001), a self-report instrument designed to examine parenting styles based on children’s and adolescents’ perceptions of their mothers’ and fathers’ behaviors. The instrument is suitable for participants aged 10–18 years and is based on a bidimensional model of parental socialization comprising two orthogonal dimensions: acceptance/involvement (warmth) and strictness/imposition. The acceptance/involvement dimension is operationalized through four parental practices—warmth (e.g., “He/she shows affection”), dialogue (e.g., “He/she talks to me”), indifference (e.g., “He/she seems indifferent”), and detachment (e.g., “It’s the same to him/her”)—with indifference and detachment being negatively related to this dimension. The strictness/imposition dimension includes three practices: revoking privileges (e.g., “He/she takes something away from me”), verbal scolding (e.g., “He/she scolds me”), and physical punishment (e.g., “He/she hits me”).

These seven parental practices are evaluated across 29 everyday family situations reflecting typical parent–adolescent interactions, of which 13 correspond to obedience contexts where adolescents comply with family norms (e.g., “If I do what he/she tells me to do”), and 16 represent disobedience contexts involving violations of family norms (e.g., “If I break or ruin something at home”). Warmth and indifference are assessed in response to obedience situations, whereas dialogue, detachment, verbal scolding, physical punishment, and revoking privileges are evaluated in disobedience situations. Adolescents report the frequency with which their mother and father use each practice using a four-point Likert scale ranging from 1 (“never”) to 4 (“always”).

The family score in acceptance/involvement (warmth) was obtained by averaging the responses in affection, dialogue, indifference, and detachment (in the last two subscales, the responsiveness were reverse-coded when negatively related to the dimension). The family score in severity/imposition (strictness) was obtained by averaging the responses in verbal coercion, physical coercion, and deprivation (Lamborn et al., 1991; Steinberg et al., 1994). Both family indices range between 1 and 4 points, corresponding to high scores, high levels of acceptance/involvement, and severity/imposition. The four parenting styles are defined by the combined effects of both warm and strict parenting practices: authoritative (warmth and strictness), indulgent (warmth without strictness), authoritarian (strictness without warmth) and neglectful (neither warmth nor strictness). Its factor structure and its invariance of the demographic variables sex and age have been confirmed in different studies (Martínez et al., 2011, 2012), as well as the orthogonality of the two main dimensions (Lim & Lim, 2003).

The factor structure of the *ESPA29* has been robustly supported by both exploratory (Martínez et al., 2011, 2012; Musitu & García, 2001) and confirmatory (Martínez et al., 2011, 2017) factor analyses across multiple studies. Originally developed and validated in Spain (Musitu & García, 2001; Martínez et al., 2019), the instrument has subsequently been translated and psychometrically validated in several languages, including English and German (Martínez et al., 2017), Portuguese (Nunes et al., 2015), and Basque (López-Jáuregui & Oliden, 2009), demonstrating its cross-cultural applicability. Owing to its solid theoretical foundation and strong psychometric properties, the *ESPA29* has been extensively employed to investigate parental socialization in relation to a wide range of developmental outcomes, including self-concept, school adjustment, behavioral problems, substance use, bullying and cyberbullying, reactive and proactive violence, child-to-parent violence, and the development of prosocial values (Fuentes et al., 2015). In the present study, the *ESPA29* exhibited excellent internal consistency. Cronbach’s alpha was 0.94 for the acceptance/involvement dimension and 0.92 for the strictness/imposition dimension. Additionally, the reliability indices for the specific parental practice subscales were consistently high, with alpha values ranging from 0.92 to 0.96, further supporting the scale’s strong internal reliability.

#### 2.3.2. Multidimensional Model of Self-Concept

The five-factor multidimensional structure of the *AF5* (F. García & Musitu, 1999) has been consistently supported through both exploratory and confirmatory (O. F. García et al., 2018; Murgui et al., 2012) factor analyses, confirming the robustness of its theoretical model. Originally developed and validated in Spain (F. García & Musitu, 1999; J. F. García et al., 2011; F. García et al., 2013; Tomás & Oliver, 2004), the *AF5* has since been translated, adapted, and validated in several languages, including English (F. García et al., 2013), Portuguese and Brazilian (O. F. García et al., 2018), Basque (Elosua & Muñiz, 2010), and Catalan (Cerrato et al., 2011). The scale has been widely applied across diverse research domains, such as academic performance and stress, self-determined motivation and well-being, interpersonal communication, bullying and cyberbullying victimization, antisocial behavior in childhood and adolescence (F. García et al., 2019), long-term socialization outcomes, and parenting and family socialization processes in both traditional and digital contexts.

In the present study, the *AF5* indicated adequate-to-good internal consistency, and Cronbach’s alpha coefficients indicated satisfactory reliability across all five dimensions: academic (0.86), social (0.68), emotional (0.74), family (0.79), and physical (0.73).

#### 2.3.3. Academic Achievement

Academic achievement was measured using students’ official final grades from the previous academic year, obtained from school records as an objective and standardized indicator of prior academic performance. It was reported using different grading systems in Spain (1–10, with 10 indicating the highest performance) and Germany (1–6, with 1 indicating the highest performance). German students’ scores were reverse-coded and rescaled to a 10-point metric to ensure comparability across samples. This approach is supported by previous research demonstrating that grades from earlier courses (e.g., cumulative GPA or final marks) are robust predictors of subsequent academic achievement across educational levels (Alyahyan & Düştegör, 2020; Dorta-Guerra et al., 2019; Nunes et al., 2023).

#### 2.3.4. Substance Use

Substance use was assessed using four indices (items), each assessing a different substance (F. García & Gracia, 2009; Lamborn et al., 1991; Sanjuan & Langenbucher, 1999). The measure of current drug use taps the frequency of involvement with alcohol, tobacco, marijuana, and other illicit drugs. Subjects provided self-report data on the frequency of use or abuse of these substances, on a 4-point Likert-type scale with four categories ranging from “never” to “often.” Greater scores indicate higher drug frequency of use. Cronbach’s alpha was 0.75.

### 2.4. Data Analysis

Parental socialization was operationalized using seven parental practices, which were combined to form the two theoretically independent dimensions of warmth and strictness. These dimensions were dichotomized at the median to classify participants into four parenting styles for each country, following established frameworks (Lamborn et al., 1991; Steinberg et al., 1994): authoritative (high warmth, high strictness), indulgent (high warmth, low strictness), authoritarian (low warmth, high strictness), and neglectful (low warmth, low strictness). Subsequently, Pearson correlation analyses were conducted to examine the general associations between parental behaviors and adolescent adjustment indicators, including self-concept, substance use, and academic achievement.

To contextualize the main analysis, we first examined a preliminary multivariate analysis of parenting practices and dimensions (2 × 2 × 2 MANOVA) by sex (male and female), by age (12–15 years and 15–17 years), and by country (German vs. Spanish samples), providing additional contextual information, although these analyses were not the main focus of this study.

Finally, to examine the influence of parenting style and national context on adolescent adjustment, a multivariate factorial approach was employed using a 4 × 2 MANOVA. Parenting style (indulgent, authoritative, authoritarian, and neglectful) and country (Spain vs. Germany) were specified as independent variables, while the dependent variables comprised multiple domains of adjustment, including self-concept dimensions, substance use, and academic achievement. When significant multivariate effects were detected, follow-up univariate analyses of variance were performed for each dependent measure. Bonferroni-adjusted post hoc tests were subsequently conducted to correct for multiple comparisons. This analytical procedure enabled a comprehensive evaluation of both main effects and interaction effects of parenting style and cultural context on adolescent outcomes.

## 3. Results

### 3.1. Family Parenting Styles

Descriptive statistics are presented in Table 1 for the parenting dimensions of warmth and strictness across each parenting style in both the Spanish and German samples. Specifically, means and standard deviations are provided for parental warmth and strictness as a function of parenting style classification. Additionally, the frequency and percentage distribution of each parenting style within each country is reported.

Post hoc analyses indicated that the two principal dimensions were relatively orthogonal (r = 0.157, R^2^ = 0.02, *p* < 0.01). Furthermore, the cross-distribution of families across the four parenting styles by child sex, *F*(3, 722) = 2.45, *p* > 0.05, and by child age, *F*(1, 724) = 2.01, *p* > 0.05, was statistically homogeneous.

### 3.2. Correlations Between the Two Dimensions of Parental Socialization, and the Five Factors of Self-Concept and Academic Performance and Substance Use

Bivariate correlations between the parental dimensions of strictness and warmth and several domains of self-concept (academic, social, emotional, family, physical, and achievement), as well as substance use are displayed (Table 2). With respect to strictness, the results revealed significant associations with several dimensions of self-concept. Strictness was negatively associated with academic (r = −0.078, *p* < 0.01), social (r = −0.110, *p* < 0.01), and emotional self-concept (r = −0.204, *p* < 0.01) and family self-concept (r = −0.292, *p* < 0.01). Although these associations reached statistical significance, the effect sizes were small, indicating limited practical relevance. Accordingly, these findings should be interpreted with caution, and strong conclusions should be avoided in the absence of meaningful practical significance. No significant correlations were observed between strictness and physical self-concept, achievement self-concept, or substance use. In contrast, warmth showed positive and statistically significant correlations with multiple dimensions of self-concept, including academic self-concept (r = 0.263, *p* < 0.01), social self-concept (r = 0.167, *p* < 0.01), emotional self-concept (r = 0.110, *p* < 0.01), family self-concept (r = 0.363, *p* < 0.01), physical self-concept (r = 0.152, *p* < 0.01), and achievement self-concept (r = 0.263, *p* < 0.01). These findings indicate that higher levels of parental warmth were consistently associated with more positive self-evaluations across diverse areas of personal and social functioning. Finally, warmth was negatively and significantly correlated with substance use (r = −0.149, *p* < 0.01), suggesting that a parental context characterized by greater emotional support and affection may function as a protective factor against risk behaviors. Overall, the results revealed differential patterns, such that parental warmth was associated with positive indicators of self-concept and lower substance use, whereas parental strictness was primarily linked to negative dimensions of self-concept, particularly within the emotional and family domains. Furthermore, analyses also showed that the two parental dimensions, warmth and strictness, consistent with the orthogonality assumption, were modestly inter-correlated (r = 0.157, R^2^ = 0.02, *p* < 0.001).

### 3.3. Preliminary Multivariate Analysis for Parenting Practices and Dimensions

A preliminary multivariate analysis of variance (MANOVA; 2 × 2 × 2) was conducted to examine parenting practices and dimensions as a function of sex (male, female) and age group (12–15 years, 15–17 years) in the German and Spanish sample, providing contextual evidence to better situate the focus of the present study (Table 3).

#### 3.3.1. Interaction Effects

The multivariate analysis revealed differential patterns for the interaction terms involving sex (A), age (B), and country (C). Specifically, the interaction between sex and age (A × B) was not statistically significant, with Λ = 0.990, *F*(9, 710) = 0.77, *p* = 0.639, and *η*^2^ = 0.010, indicating that the effect of sex on the set of dependent variables did not vary as a function of age. The interaction between age and country (B × C) did not reach statistical significance, with Λ = 0.980, *F*(9, 710) = 1.60, *p* = 0.110, and *η*^2^ = 0.020, suggesting that age-related differences in parental socialization practices were comparable across the German and Spanish sample. The three-way interaction among sex, age, and country (A × B × C) was also not statistically significant (Λ = 0.988, *F*(9, 710) = 0.95, *p* = 0.484, *η*^2^ = 0.012). This result indicates that the combined effects of gender and age on parenting practices do not differ across countries, pointing to a stable pattern of gender-by-country differences that is consistent across age groups. In contrast, a statistically significant interaction emerged between sex and country (A × C) (Λ = 0.995, *F*(9, 710) = 3.69, *p* < 0.001, *η*^2^ = 0.045). This finding indicates that gender differences in parental socialization practices are contingent upon the national context. Although the effect size was small to moderate, the interaction suggests meaningful cross-national variation in how sons and daughters are differentially treated, warranting further examination through follow-up univariate and simple effects analyses.

**Figure 2:**
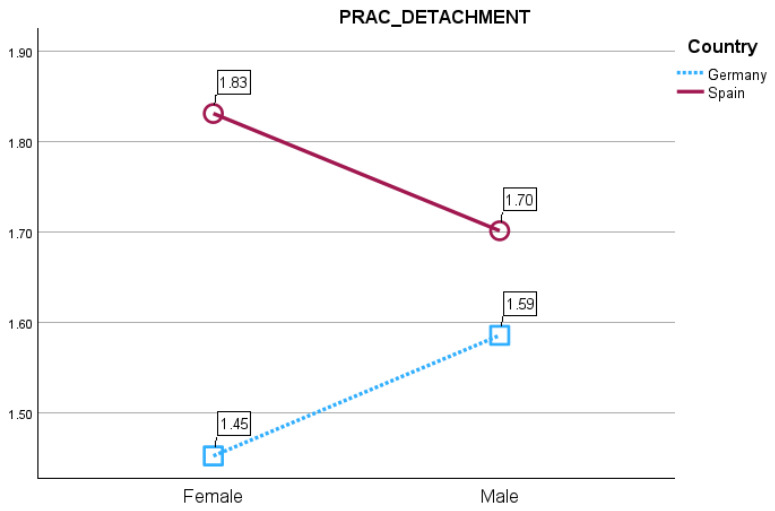
Interaction between sex and country for parental detachment practice.

The practice of detachment, defined as parental indifference or non-responsiveness to norm-violating behavior, showed significant differences. In the Spanish sample, parents reported higher levels of detachment toward daughters (*M* = 1.59, *SD* = 0.03) than sons (*M* = 1.57, *SD* = 0.03) (*F*(1, 718) = 6.01, *p* = 0.014, *η*^2^_p_ = 0.008) (Figure 2). This pattern was reversed in the German sample, where detachment was more pronounced toward sons (*M* = 1.61, *SD* = 0.03) than daughters (*M* = 1.58, *SD* = 0.03) (*F*(1, 718) = 7.87, *p* = 0.005, *η*^2^_p_ = 0.011).

**Figure 3:**
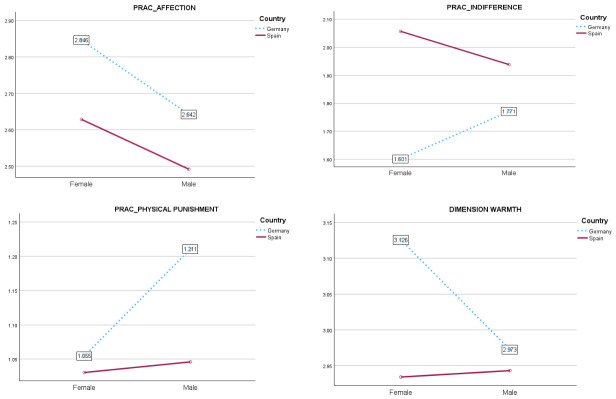
Interaction between sex and country (single effects in the German sample) for parental affection, indifference, physical punishment practices, and warmth dimension.

No additional significant sex differences were observed in the Spanish sample across the other parenting practices and socialization dimensions examined (all *p* > 0.05), suggesting relative uniformity in the treatment of sons and daughters within this cultural context. In contrast, the German sample revealed a consistent pattern of sex-based differentiation across multiple parenting dimensions. As illustrated by the graphs in Figure 3, German parents reported significantly higher levels of affection toward daughters (*M* = 2.84, *SD* = 0.04) than sons (*M* = 2.71, *SD* = 0.04) (*F*(1, 718) = 7.47, *p* = 0.006, *η*^2^_p_ = 0.010). Convergent findings emerged for the dimension of warmth, which was also significantly elevated in interactions with daughters (*M* = 3.12, *SD* = 0.05) relative to sons (*M* = 2.94, *SD* = 0.05) (*F*(1, 718) = 10.49, *p* = 0.001, *η*^2^_p_ = 0.014). Conversely, negative parenting practices were more frequently directed toward sons. Indifference was significantly higher for sons (*M* = 1.63, *SD* = 0.03) than daughters (*M* = 1.58, *SD* = 0.03) (*F*(1, 718) = 6.76, *p* = 0.010, *η*^2^_p_ = 0.009), consistent with the detachment findings reported above. Most pronounced was the disparity in physical punishment, which was substantially more prevalent in the parenting of sons (*M* = 1.72, *SD* = 0.04) compared to daughters (*M* = 1.48, *SD* = 0.04) (*F*(1, 718) = 48.47, *p* < 0.001, *η*^2^_p_ = 0.063), representing a medium-to-large effect.

Taken together, these findings indicate that, while Spanish parenting practices remain largely undifferentiated by child gender, German parents exhibit systematic differentiation, characterized by greater affective warmth toward daughters and increased disciplinary control—particularly physical punishment—toward sons.

#### 3.3.2. Main Effects of Sex, Age, and Country on Parenting Practices and Dimensions

A primary effect of sex was found (Table 4), revealing statistically significant differences in warmth, with *F*(1, 718) = 4.16, *p* < 0.05, with parents reporting higher levels toward daughters (*M* = 3.03, *SE* = 0.02) than sons (*M* = 2.96, *SE* = 0.03). A significant effect was also observed for affection, with *F*(1, 718) = 9.33, *p* < 0.01, indicating that female children received significantly higher levels of affection (*M* = 2.74, *SE* = 0.04) compared to male children (*M* = 2.57, *SE* = 0.04). Regarding physical punishment, with *F*(1, 718) = 26.17, *p* < 0.001, the results showed that female children received significantly lower levels (*M* = 1.04, *SE* = 0.01) compared to male children (*M* = 1.13, *SE* = 0.01).

The significant main effects of adolescent age emerged across multiple practices and dimensions of parenting (Table 4), revealing systematic developmental shifts in how parents socialize younger (12–15 years) versus older (16–19 years) adolescents. Parents reported significantly higher levels of overall Warmth toward younger adolescents (*M* = 3.09, *SD* = 0.03) than toward older adolescents (*M* = 2.90, *SD* = 0.02) (*F*(1, 718) = 27.31, *p* < 0.001). For the practice of Affection, younger adolescents receiving substantially more affection (*M* = 2.81, *SD* = 0.04) compared to older ones (*M* = 2.49, *SD* = 0.04) (*F*(1, 718) = 33.21, *p* < 0.001). In contrast, no significant age differences emerged for Dialog, indicating that parental communicative exchange remains relatively stable across adolescence. Indifference was significantly higher among older adolescents (*M* = 1.96, *SD* = 0.03) than younger adolescents (*M* = 1.72, *SD* = 0.04) (*F*(1, 718) = 24.55, *p* < 0.001). Similarly, Detachment was more pronounced in the older age group (*M* = 1.71, *SD* = 0.02) compared to the younger group (*M* = 1.57, *SD* = 0.03) (*F*(1, 718) = 16.22, *p* < 0.001). Significant age differences also emerged within the strictness dimension. Verbal Scolding was more frequently directed toward younger adolescents (*M* = 2.25, *SD* = 0.03) than older adolescents (*M* = 2.01, *SD* = 0.03) (*F*(1, 718) = 30.46, *p* < 0.001). Revocation of Privileges followed a similar pattern, with younger adolescents receiving higher levels (*M* = 1.59, *SD* = 0.03) compared to older adolescents (*M* = 1.35, *SD* = 0.02) (*F*(1, 718) = 42.78, *p* < 0.001). However, no significant age difference emerged for Physical Punishment, with *F*(1, 718) = 2.81, *p* > 0.05, suggesting that this disciplinary practice does not vary systematically across the two age groups. Taken together, these findings reveal a coherent developmental trajectory in which younger adolescents experience greater parental warmth, affection, and disciplinary intervention, while older adolescents encounter increased emotional distancing and reduced control, likely reflecting parents’ adaptation to their children’s growing autonomy and developmental needs.

A series of one-way ANOVAs was conducted to examine the main effects of country (German vs. Spanish sample) on parenting practices and dimensions (Table 4). Significant cross-national differences emerged across multiple indicators of both warmth and strictness, revealing distinct patterns of parental socialization in each cultural context. Within the warmth dimension, significant country differences were observed for two practices. German parents reported significantly higher levels of Dialog (*M* = 2.72, *SD* = 0.03) than their Spanish counterparts (*M* = 2.52, *SD* = 0.03) (*F*(1, 718) = 18.47, *p* < 0.001). In contrast, Spanish parents exhibited significantly higher levels of Indifference (*M* = 1.92, *SD* = 0.03) compared to German parents (*M* = 1.76, *SD* = 0.03) (*F*(1, 718) = 12.34, *p* < 0.001). No significant main effects of country emerged for overall Warmth, Affection, or Detachment (all *p* > 0.05), indicating comparable levels of these practices across both nations. Regarding the dimension of strictness and its practices, differences were observed between countries. German parents reported significantly higher levels of Verbal Scolding (*M* = 2.24, *SD* = 0.03) than Spanish parents (*M* = 2.02, *SD* = 0.03) (*F*(1, 718) = 26.78, *p* < 0.001). This pattern was consistent for Physical Punishment, with German parents reporting higher levels (*M* = 1.14, *SD* = 0.01) compared to Spanish parents (*M* = 1.03, *SD* = 0.01) (*F*(1, 718) = 38.92, *p* < 0.001). Similarly, German parents reported significantly higher levels of Revocation of Privileges (*M* = 1.56, *SD* = 0.02) than Spanish parents (*M* = 1.38, *SD* = 0.02) (*F*(1, 718) = 32.15, *p* < 0.001).

### 3.4. Effects of Parenting Styles and Country on Adolescent Adjustment

A multivariate analysis of the effects of parenting styles (indulgent, authoritative, authoritarian, and neglectful) and country (Germany and Spain) on the adjustment of adolescents (academic self-concept, social self-concept, emotional self-concept, family self-concept, physical self-concept, and academic achievement and substance use) was performed, the results of which are shown in Table 5.

#### 3.4.1. Interaction Effects

The multivariate analysis revealed a significant parenting style × country interaction, with Wilks’ Λ = 0.885, *F*(21, 2045) = 4.23, *p* < 0.001, *η*^2^ = 0.04, indicating that the associations between parenting styles and adolescent adjustment varied across national contexts. Follow-up univariate analyses showed that this interaction was significant for Academic Self-Concept (*F*(3, 718) = 4.32, *p* = 0.005), *η*^2^ = 0.018; Academic Achievement (*F*(3, 718) = 14.22, *p* < 0.001, *η*^2^ = 0.056); and Substance Use (*F*(3, 718) = 2.97, *p* = 0.031, *η*^2^ = 0.012), but not for Social, Emotional, Family, or Physical Self-Concept (all *ps* ≥ 0.354). These findings require us to look at simple effects.

**Figure 4:**
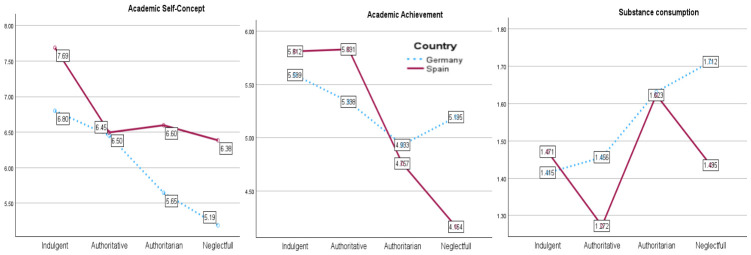
Interaction between parenting styles and country for academic self-concept, academic achievement, and substance consumption.

Regarding Academic Self-Concept, the pattern differed across countries, as can be observed in the graph presented in Figure 4. In the German sample, indulgent (*M* = 6.80, *SD* = 1.84) and authoritative parenting (*M* = 6.45, *SD* = 1.51) were associated with higher scores than neglectful parenting (*M* = 5.19, *SD* = 1.51), and indulgent parenting also exceeded authoritarian parenting (*M* = 5.65, *SD* = 1.86). In Spain, indulgent parenting (*M* = 7.69, *SD* = 1.98) was associated with significantly higher academic self-concept than authoritative (*M* = 6.50, *SD* = 1.74), authoritarian (*M* = 6.60, *SD* = 1.92), and neglectful parenting (*M* = 6.39, *SD* = 1.64), which did not differ from one another. Cross-national comparisons indicated higher scores in the Spanish sample than German sample within the indulgent, authoritarian, and neglectful groups, but not within the authoritative group.

In terms of Academic Achievement, indulgent (*M* = 5.59, *SD* = 1.36) and authoritative parenting (*M* = 5.34, *SD* = 1.11) were associated with a higher performance than authoritarian parenting (*M* = 4.93, *SD* = 1.37) in the German sample, whereas neglectful parenting (*M* = 5.20, *SD* = 1.11) did not differ from the former two. In the sample of Spanish adolescents, both indulgent (*M* = 5.81, *SD* = 1.45) and authoritative parenting (*M* = 5.83, *SD* = 1.28) were associated with higher achievement than authoritarian (*M* = 4.76, *SD* = 1.41) and neglectful parenting (*M* = 4.16, *SD* = 1.21), with the neglectful group showing the lowest performance. Spanish adolescents outperformed German adolescents in the authoritative group, whereas the reverse pattern emerged in the neglectful group, as can be observed in Figure 4.

Regarding Substance Use, in the sample of German adolescents, neglectful (*M* = 1.71, *SD* = 0.53) and authoritarian parenting (*M* = 1.63, *SD* = 0.64) were associated with higher consumption than indulgent parenting (*M* = 1.42, *SD* = 0.64), with authoritative parenting (*M* = 1.46, *SD* = 0.53) occupying an intermediate position. In the Spanish sample, authoritative parenting (*M* = 1.27, *SD* = 0.60) was associated with the lowest levels of substance use, differing from authoritarian (*M* = 1.62, *SD* = 0.67) and neglectful parenting (*M* = 1.44, *SD* = 0.57); indulgent parenting (*M* = 1.47, *SD* = 0.69) did not differ from neglectful parenting. Cross-nationally, German adolescents reported higher substance use within the authoritative and neglectful groups, with no differences in the indulgent or authoritarian groups. Overall, the findings demonstrate that the implications of parenting styles for adolescents’ academic adjustment and substance use are contingent upon the national context, with indulgent parenting showing more favorable outcomes in the Spanish context and more differentiated patterns emerging in Germany.

#### 3.4.2. Main Effects of Country and Parenting Styles

Univariate analyses of variance (ANOVAs) were performed to examine the differences in adolescent adjustment by country (Germany vs. Spain) and parenting style (indulgent, authoritative, authoritarian, and neglectful) based on the adjustment factors assessed. Table 6 shows the means and standard deviations for each outcome by country and parenting style, together with the corresponding F-statistics.

**Figure 5:**
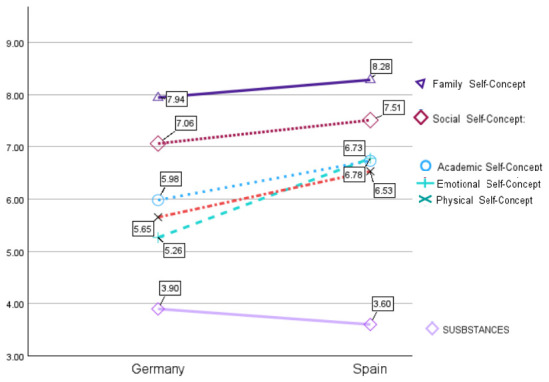
Means for adjustment factors of adolescents by countries. (Note: The values in the substance use graph were rescaled to a 10-point scale because the original scale consisted of 4 points).

Univariate analyses of variance (ANOVAs) were conducted to examine the main effect of country on the different dimensions of adjustment assessed, comparing adolescents from Germany and Spain (Figure 5). The results indicated that Spanish adolescents (*M* = 6.79, *SD* = 0.10) reported significantly higher academic self-concept scores than German adolescents (*M* = 6.02, *SD* = 0.09) (*F*(1, 718) = 35.06, *p* < 0.001). Similarly, significant differences were found in social self-concept, with Spanish participants (*M* = 7.52, *SD* = 0.09) scoring higher than their German counterparts (*M* = 7.06, *SD* = 0.08) (*F*(1, 718) = 15.19, *p* < 0.001). Regarding emotional self-concept, Spanish adolescents (*M* = 6.78, *SD* = 0.10) obtained substantially higher scores compared to German adolescents (*M* = 5.27, *SD* = 0.10), representing the largest difference observed, with *F*(1, 718) = 114.43, *p* < 0.001. In addition, family self-concept was significantly higher among Spanish participants (*M* = 8.28, *SD* = 0.09) than among German participants (*M* = 7.90, *SD* = 0.08) (*F*(1, 718) = 10.02, *p* < 0.01). The differences were also significant for physical self-concept, with Spanish adolescents (*M* = 6.53, *SD* = 0.10) reporting higher scores than German adolescents (*M* = 5.64, *SD* = 0.09) (*F*(1, 718) = 41.66, *p* < 0.001). In contrast, no statistically significant differences were found in academic achievement between the Spanish sample (*M* = 5.14, *SD* = 0.07) and German sample (*M* = 5.26, *SD* = 0.07) (*F*(1, 718) = 1.64, *p* > 0.05). Finally, substance use scores were significantly higher among German adolescents (*M* = 1.55, *SD* = 0.03) compared to Spanish adolescents (*M* = 1.45, *SD* = 0.03) (*F*(1, 718) = 5.22, *p* < 0.05).

**Figure 6:**
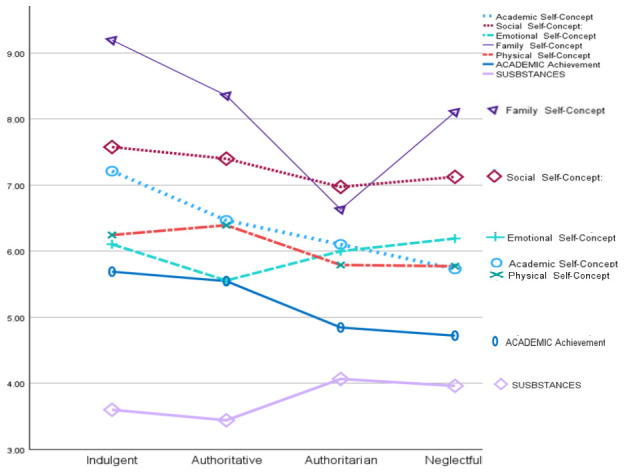
Adjustment of the adolescents according to the parenting styles. (The values in the substance use graph were rescaled to a 10-point scale because the original scale consisted of 4 points).

Univariate analyses of variance (ANOVAs) were conducted to examine the main effects of parenting style (indulgent, authoritative, authoritarian, and neglectful) on the different dimensions of adolescent adjustment assessed (Figure 6). The results revealed significant differences in academic self-concept as a function of parenting style, with *F*(3, 718) = 22.33, *p* < 0.001. Adolescents raised under an indulgent parenting style (*M* = 7.24, *SD* = 0.14) reported significantly higher academic self-concept scores than those socialized under authoritative (*M* = 6.47, *SD* = 0.12), authoritarian (*M* = 6.12, *SD* = 0.14), and neglectful styles (*M* = 5.79, *SD* = 0.12). In addition, authoritative parenting was associated with higher scores than neglectful parenting. Significant differences were also found in social self-concept, with *F*(3, 718) = 5.00, *p* < 0.01. Adolescents from indulgent families (*M* = 7.59, *SD* = 0.13) scored higher than those from authoritarian (*M* = 6.98, *SD* = 0.13) and neglectful households (*M* = 7.15, *SD* = 0.11). Furthermore, authoritative parenting (*M* = 7.43, *SD* = 0.11) yielded significantly higher social self-concept scores than authoritarian parenting. Regarding emotional self-concept, the effect of parenting style was also significant, with *F*(3, 718) = 4.17, *p* < 0.01; however, post hoc comparisons indicated only one significant difference, with neglectful parenting (*M* = 6.26, *SD* = 0.13) showing higher scores than authoritative parenting (*M* = 5.65, *SD* = 0.13). For family self-concept, a highly significant effect emerged, with *F*(3, 718) = 66.79, *p* < 0.001. Adolescents raised in indulgent families (*M* = 9.21, *SD* = 0.13) reported higher family self-concept than those raised under authoritative (*M* = 8.36, *SD* = 0.11), authoritarian (*M* = 6.65, *SD* = 0.13), and neglectful styles (*M* = 8.13, *SD* = 0.11). Additionally, authoritative parenting was associated with higher scores than authoritarian parenting, and neglectful parenting also exceeded authoritarian parenting. Significant differences were observed in physical self-concept (*F*(3, 718) = 5.76, *p* < 0.001), with authoritative parenting (*M* = 6.45, *SD* = 0.13) producing higher scores than both authoritarian (*M* = 5.81, *SD* = 0.15) and neglectful parenting (*M* = 5.82, *SD* = 0.12). Academic achievement also differed significantly across parenting styles, with *F*(3, 718) = 30.12, *p* < 0.001. Adolescents socialized under indulgent (*M* = 5.70, *SD* = 0.10) and authoritative parenting (*M* = 5.59, *SD* = 0.09) achieved a significantly higher academic performance than those raised under authoritarian (*M* = 4.85, *SD* = 0.10) and neglectful styles (*M* = 4.68, *SD* = 0.09). Finally, substance consumption differed significantly by parenting style, with *F*(3, 718) = 7.38, *p* < 0.001. Adolescents raised under an authoritarian style (*M* = 1.63, *SD* = 0.05) reported significantly higher substance use than those from indulgent (*M* = 1.44, *SD* = 0.05) and authoritative households (*M* = 1.36, *SD* = 0.04). Moreover, adolescents raised under neglectful parenting (*M* = 1.57, *SD* = 0.04) showed higher substance consumption than those raised under authoritative parenting.

Overall, these findings suggest that indulgent and authoritative parenting styles are generally associated with more positive self-concept outcomes and higher academic achievement, whereas authoritarian and neglectful styles tend to be linked to poorer adjustment indicators and, in the case of authoritarian parenting, higher levels of substance use.

## 4. Discussion

The present study addresses a central question in contemporary developmental psychology: the extent to which the association between parenting styles and adolescent adjustment reflects universal developmental principles or is culturally mediated. By comparing German and Spanish samples of adolescents—two European countries that differ substantially in levels of individualism, family cohesion, and educational values—the findings provide empirical evidence that qualifies the previous conclusions derived from early research conducted predominantly in Anglo-Saxon contexts (Baumrind, 1971; Lamborn et al., 1991; Steinberg et al., 1994). The results suggest that the effectiveness of parenting styles cannot be adequately understood independently of the normative and cultural frameworks in which they are embedded, thereby supporting a contextualist perspective that recognizes multiple, equally adaptive pathways of socialization depending on sociocultural context (Chao, 1994; Lansford, 2022; Pinquart & Kauser, 2018).

With regard to the first hypothesis, which predicted a significant association between parenting styles and adolescent adjustment, the results supported this expectation, although several nuances require careful consideration. Overall, parenting styles characterized by high warmth—indulgent and authoritative—were associated with higher levels of academic, social, family, and physical self-concept, as well as higher academic performance and lower substance use (Calafat et al., 2014; Cuadri et al., 2025; Fuentes et al., 2015; O. F. García et al., 2020; J. R. García-Perales et al., 2019; Gorostiaga et al., 2019; Kerr & Stattin, 2000; Navarro García, 2014; Rodrigues et al., 2013). In contrast, low-warmth styles—authoritarian and neglectful—were linked to poorer adjustment outcomes. These results align with the recent literature highlighting affective support as a fundamental component of psychosocial well-being across cultural contexts (Martínez et al., 2019; Pinquart & Gerke, 2019; Wei et al., 2025; Zhang et al., 2026).

However, the role of strictness—defined as behavioral control and disciplinary demandingness—proved more complex and culturally contingent, providing support for the third hypothesis concerning cross-national variation in parenting effects. In the German sample, the combination of warmth and strictness (authoritative parenting) was associated with outcomes comparable to those observed for the indulgent style across most adjustment indicators and suggested particular patterns in preventing substance use. This pattern is consistent with recent German studies documenting the effectiveness of parenting practices that integrate emotional support with structured control within a cultural context that strongly values autonomy and individual self-regulation (Azman et al., 2021; Gniewosz & Gniewosz, 2025; Ostner & Stolberg, 2015; Walper & Kreyenfeld, 2022). Longitudinal research further indicates that parenting characterized by high levels of support and supervision predicts more favorable mental health trajectories among German adolescents, even in the presence of risk factors (Kassis et al., 2025). In the Spanish sample, by contrast, the indulgent style (high warmth, low strictness) emerged as a context-dependent parenting strategy, equaling or scoring stronger than the authoritative style across nearly all assessed dimensions. This finding reinforces a well-established tradition of research demonstrating the effectiveness of indulgent parenting in Mediterranean and Latin American contexts (F. García & Gracia, 2009, 2010, 2014; Martínez & García, 2007; Rodrigues et al., 2013; Pérez-Gramaje et al., 2020). Importantly, the present results indicate that these cultural differences persist even in the twenty-first century within an increasingly globalized European environment. Previous cross-cultural research including samples from Spain, Germany, the United States, and Brazil had already identified the emergence of a “third stage” in parental socialization, which may be more congruent with indulgent parenting in cultures where family relationships represent a central social value (F. García et al., 2019).

Self-concept is the domain where cultural differences are the most salient. Self-concept, particularly its academic and emotional dimensions, proved especially sensitive to the interaction between parenting style and cultural context. Spanish adolescents raised in indulgent families reported the highest levels of academic self-concept, significantly outperforming peers from authoritative, authoritarian, and neglectful families. This finding supports the notion that, in contexts characterized by strong family cohesion and emotional closeness, expressions of warmth alone may foster positive academic self-perceptions, without additional disciplinary demandingness providing incremental benefits (F. García & Gracia, 2014; Martínez et al., 2019). Research conducted with Italian adolescents—a culturally comparable Mediterranean context—similarly found that parental support was positively associated with psychological well-being, whereas psychological control showed negative associations, reinforcing the view that warmth constitutes the primary active ingredient in adaptive socialization processes (Inguglia et al., 2016). In the German sample, however, indulgent and authoritative parenting yielded comparable levels of academic self-concept; both showed higher scores than those associated with authoritarian and neglectful styles. This pattern may reflect culturally specific conceptions of academic success and its developmental antecedents. In contexts emphasizing self-regulation and individual responsibility, moderate parental demandingness may be interpreted by adolescents as an expression of involvement and concern, thereby contributing positively to academic self-perceptions (Gniewosz & Gniewosz, 2025; Juang & Silbereisen, 1999). Recent findings further indicate that German adolescents value a balance between emotional support and clearly defined limits, consistent with the observed effectiveness of authoritative parenting in this context (Mandelkow, 2025).

Particularly striking were the cross-national differences in emotional self-concept. Spanish adolescents reported substantially higher scores than German adolescents regardless of parenting style, suggesting the influence of broader cultural factors shaping the emotional expression and perceptions of well-being. Previous cross-cultural studies have shown that Mediterranean societies tend to emphasize emotional expressiveness and interpersonal closeness, potentially fostering greater emotional awareness and more positive emotional self-evaluations (Zhang et al., 2026; Olivari et al., 2015). Alternatively, these differences may reflect culturally shaped response styles or normative expectations regarding emotional adjustment, an issue that future research should address through formal tests of factorial invariance.

The academic performance domain reveals the shared patterns and context-specific differences. In the domain of academic performance, both countries converged in identifying high-warmth parenting styles—indulgent and authoritative—as the most beneficial, whereas low-warmth styles—authoritarian and neglectful—were associated with less favorable outcomes. This pattern is consistent with numerous studies demonstrating moderate positive associations between parental warmth and academic achievement across diverse cultural contexts, thereby providing additional support for the first hypothesis (Aunola et al., 2000; Newman et al., 2015; Pinquart & Kauser, 2018; Torres-Villa, 1995; Wei et al., 2025). Nevertheless, a notable difference emerged, again supporting the third hypothesis: whereas, in the Spanish sample, the neglectful style was associated with the lowest academic performance, in the German sample, it did not significantly differ from the authoritative style. This divergence may be interpreted in light of differences in the educational systems and culturally normative expectations regarding parental involvement. In Spain, where strong family involvement in schooling is traditionally expected, parental disengagement may have particularly detrimental consequences. In contrast, the German educational system promotes early student autonomy and individual responsibility (Dorta-Guerra et al., 2019; Alyahyan & Düştegör, 2020), potentially reducing the relative influence of parental involvement. Recent research emphasizes that academic achievement is shaped by multiple interacting factors beyond parenting style, including socioeconomic status, school climate, and individual student characteristics (Yang et al., 2024). Accordingly, the present findings should be interpreted cautiously, as relevant control variables were not included in the analyses.

The substance use domain shows context-dependent protective effects. Substance use represented one of the adjustment domains in which the interaction between parenting style and cultural context was most clearly observed, providing strong support for the third hypothesis. In the Spanish sample, authoritative parenting was associated with the lowest levels of substance use, whereas, in the German sample, both indulgent and authoritative styles exerted comparable protective effects. These findings suggest that parental control may carry distinct cultural meanings across contexts. In Spain, where parental monitoring may be interpreted as an expression of care within a relationally oriented cultural framework, its combination with warmth appears particularly suitable in preventing risk behaviors (Calafat et al., 2014). In the German sample, however, the absence of strict control does not appear to increase risk when warmth is present, suggesting that emotional support alone may function as a sufficient protective factor in contexts where adolescent autonomy is more strongly institutionalized. These results are consistent with previous research documenting the protective role of parental warmth against substance use across European countries (Adalbjarnardottir & Hafsteinsson, 2001; Calafat et al., 2014). Of concern, however, is the relatively high prevalence of substance use among German adolescents raised under authoritarian and neglectful parenting, underscoring the need for targeted interventions aimed at families exhibiting these profiles (Kassis et al., 2025; Universitätsklinikum Ulm & UNICEF Deutschland, 2025).

Next, we examine the variations in parenting practices in relation to sex, age, and country. Regarding the second hypothesis—which predicted the variations in parenting practices as a function of adolescents’ sex, age, and country—the findings revealed a nuanced pattern. Multivariate analyses showed the significant main effects of sex, age, and country on parenting dimensions, partially confirming this hypothesis. Daughters received higher levels of warmth and affection than sons, whereas sons experienced higher levels of physical punishment, consistent with research documenting gender-differentiated socialization processes (Zhang et al., 2026). Younger adolescents (12–15 years) received higher levels of warmth, affection, and disciplinary strategies than older adolescents (16–19 years), reflecting parental adaptation to increasing adolescent autonomy (Steinberg, 2005). Significant cross-national differences were also observed, with German parents reporting higher levels of dialogue, reprimands, physical punishment, and withdrawal of privileges compared with Spanish parents. Most notably, a significant interaction between sex and country indicated that gender differences in parenting practices are culturally contingent rather than universal. Whereas minimal gender differentiation was observed in the Spanish sample—suggesting relatively uniform socialization—the German sample displayed a consistent pattern of greater warmth toward daughters and higher physical punishment toward sons. This finding aligns with recent evidence indicating the persistence of gender-differentiated disciplinary practices despite policy reforms and awareness campaigns (Hein et al., 2025; Öztürk et al., 2023). Research further suggests that such differentiated practices may influence the development of non-cognitive skills and gender identity formation (Zhang et al., 2026). Future studies should examine how these gendered parenting patterns translate into differential adjustment outcomes across cultural contexts.

The present findings should be interpreted in light of prior research that has frequently linked indulgent parenting to less favorable outcomes, particularly in self-regulation and behavioral adjustment (Lansford et al., 2018; Kuppens & Ceulemans, 2019; Pinquart, 2017). This apparent discrepancy may reflect contextual and methodological factors. In Southern European contexts, high parental warmth combined with lower behavioral control may not have the same implications observed in other cultural settings. Additionally, differences in the operationalization of parenting styles and the use of categorical classifications may contribute to the variability across studies. It is also possible that indulgent parenting is associated with positive outcomes in some domains but less optimal functioning in others, leading to mixed findings. Overall, the results suggest that the effects of parenting styles are context-dependent and domain-specific.

### 4.1. Theoretical and Practical Implications

From a theoretical perspective, the findings contribute to the longstanding debate concerning the universality versus cultural specificity of parenting models. The results clearly support the view that parenting adjustment is culturally mediated (Deater-Deckard & Dodge, 1997; Pinquart & Kauser, 2018), challenging earlier claims regarding the universal variation across contexts of the authoritative style. Beyond this, the findings suggest a conceptual distinction between universal and culturally contingent components of parenting: parental warmth appears to constitute a universally beneficial element—partially confirming the first hypothesis at its most general level—whereas strictness operates as a culturally moderated factor whose adjustment depends on its contextual meaning, thereby fully supporting the third hypothesis. This distinction may inform the development of more integrative theoretical models capable of reconciling universal developmental processes with cultural variability (Grusec & Goodnow, 1994; Grusec et al., 2000, Darling & Steinberg, 1993; Morris et al., 2021).

At the applied level, the findings have important implications for the design of family-based interventions and prevention programs. First, they underscore the need for culturally sensitive interventions aligned with the norms and values of the contexts in which they are implemented. Programs promoting authoritative parenting may be well-aligned with the German context, while showing a different pattern of applicability in Spain, where indulgent parenting demonstrates comparative higher scores. Second, given the consistent protective role of parental warmth across contexts, interventions should prioritize the development of parenting competencies related to emotional expression, communication, and supportive engagement. Finally, the persistence of gender-differentiated disciplinary practices in the German sample—qualifying the scope of the second hypothesis—highlights the need for targeted initiatives promoting egalitarian and gender-sensitive parenting approaches.

### 4.2. Limitations and Future Directions

Several limitations should be considered when interpreting the findings of this study. First, the cross-sectional design precludes causal inferences regarding the directionality of the associations between parenting styles and adolescent adjustment. Although prior research generally supports a pathway from parenting to adjustment, reciprocal influences and the potential role of unmeasured third variables cannot be ruled out (Steinberg, 2001). Longitudinal designs would be necessary to examine developmental trajectories and to better disentangle causal processes over time (Gniewosz & Gniewosz, 2025).

Second, the reliance on adolescent self-reports may introduce common-method variance and shared reporter bias. While adolescents’ perceptions are central for understanding how parenting practices are experienced and internalized (Grusec & Goodnow, 1994), the exclusive use of a single informant may inflate associations among variables. Future research would benefit from incorporating multiple informants, including parents and teachers, as well as complementary methodological approaches, in order to obtain a more comprehensive and reliable assessment (Pérez-Gramaje et al., 2020; Yang et al., 2024).

Third, although sex and age were statistically controlled, other relevant contextual and sociodemographic factors were not included in the analyses. Variables such as socioeconomic status, family structure, migration background, and peer relationships may act as moderators or confounding factors, potentially shaping both parenting processes and adolescent adjustment (Öztürk et al., 2023). The omission of these variables may limit the interpretability and generalizability of the findings.

Fourth, the operationalization of parenting styles based on dimensional cut-offs may entail a loss of information and impose artificial categorical distinctions on inherently continuous constructs. This classification approach may obscure the within-group variability and affect the robustness of comparisons between parenting styles.

Finally, the sample was drawn from specific school contexts, which raises the possibility of sample selectivity and school-level or cohort effects. As a result, the extent to which the findings can be generalized to broader populations remains uncertain. Taken together, these limitations suggest that the results should be interpreted with caution and underscore the need for future research employing longitudinal designs, multi-informant data, and more refined analytic strategies.

Future research should address the factorial invariance of parenting styles across cultural contexts to ensure that measurement models operate equivalently and allow valid cross-cultural comparisons. In addition, the limited inclusion of potential confounding variables represents a methodological constraint, as factors such as socioeconomic variation, parental education, school type, migration background, family size, and school-level effects were not fully considered beyond sex, age, and country.

Finally, future research should investigate the mechanisms underlying cross-cultural differences by incorporating direct measures of cultural values, perceived parenting norms, and internalization processes (Grusec & Goodnow, 1994; Darling & Steinberg, 1993; Inguglia et al., 2016). Despite these limitations, this study provides robust evidence on the interaction between parenting styles and cultural context in shaping adolescent adjustment, drawing on representative samples from two European countries with distinct cultural profiles. The findings largely support the three proposed hypotheses, underscoring the need to move beyond universalistic models toward a contextualist framework that acknowledges multiple equally effective forms of parenting—a perspective that is both theoretically more accurate and practically essential for designing culturally sensitive interventions.

## 5. Conclusions

Overall, the accumulated evidence supports several key conclusions. First, the association between parenting styles and adolescent adjustment is shaped by cultural context, challenging the assumption that the authoritative style represents a traditionally accepted framework (F. García et al., 2019; Pinquart & Kauser, 2018). Second, within European settings, the indulgent style—characterized by high warmth and lower strictness—has been found to perform as well as or even higher than the authoritative style across major indicators of adjustment, including self-concept, academic performance, and substance use (Calafat et al., 2014; F. García & Gracia, 2009, 2010; Gimenez-Serrano et al., 2022). Third, the influence of parenting styles appears to extend beyond adolescence, with long-term effects observable across the life span and even across generations (Gimenez-Serrano et al., 2022; F. García et al., 2019). Fourth, gender differences are evident both in adolescents’ exposure to parental practices and in the developmental impact of those practices (Zhang et al., 2026).

These findings underscore the importance of continuing cross-cultural and comparative research on parenting styles and adolescent adjustment, particularly within European populations. They also carry practical implications for family-based interventions and parenting policies, highlighting the need for culturally responsive approaches that recognize the variability in the effectiveness of parental practices across sociocultural contexts (Yang et al., 2024).

Finally, the present study is particularly relevant as it calls into question the universal applicability of the authoritative parenting style, traditionally defined by high warmth and high strictness and predominantly supported within Anglo-Saxon research traditions. The findings indicate that, in Germany, as in other European and Latin American contexts, the indulgent style may be equally or even more appropriate for certain indicators of adolescent adjustment. Overall, these results are consistent with a growing body of literature suggesting that the effects of parenting styles are not universal but depend on the cultural and contextual conditions in which they are embedded.

## Figures and Tables

**Table 1:** Distribution of participants according to parenting style, and mean score and standard deviation on parenting dimensions for each country.

	Total	Indulgent	Authoritative	Authoritarian	Neglectful
Total *n* (%)	726 (100)	148 (20.4)	208 (28.7)	151 (20.8)	219 (30.2)
Spanish sample					
*n* (%)	331	69 (20.8)	89 (26.9)	73 (22.1)	100 (30.2)
Warmth: *M* (*SD*)	2.93 (0.46)	3.33 (0.26)	3.31 (0.19)	2.56 (0.28)	2.58 (0.35)
Strictness: *M* (*SD*)	1.39 (0.26)	1.18 (0.12)	1.57 (0.17)	1.65 (0.22)	1.19 (0.11)
German sample					
*n* (%)	395	79 (20)	119 (30.1)	78 (19.7)	119 (30.1)
Warmth: *M* (*SD*)	3.0 (0.48)	3.47 (0.17)	3.48 (0.19)	2.67 (0.78)	2.57 (0.27)
Strictness: *M* (*SD*)	1.7 (0.41)	1.37 (0.16)	2.02 (0.21)	2.13 (0.27)	1.34 (0.13)

**Table 2:** Correlations between parenting dimensions, self-concept factors, academic achievement, and drug use.

	1	2	3	4	5	6	7	8
1. Strictness								
2. Warmth	0.157 **							
3. Academic SC	−0.078 *	0.263 **						
4. Social SC	−0.110 **	0.167 **	0.222 **					
5. Emotional SC	−0.204 **	−0.110 **	−0.007	0.328 **				
6. Family SC	−0.292 **	0.363 **	0.396 **	0.296 **	0.142 **			
7. Physical SC	−0.063	0.152 **	0.380 **	0.454 **	0.230 **	0.310 **		
8. Achievement	0.025	0.263 **	0.296 **	0.087 *	−0.059	0.264 **	0.240 **	
9. Substances use	0.019	−0.149 **	−0.147 **	0.068	−0.004	−0.139 **	−0.046	−0.039

* *p* < 0.05. ** *p* < 0.01. Effect size (r = 0.10, R^2^ = η^2^ = 0.01): small, r ≈ 0.10; medium, r ≈ 0.30; and large, r ≈ 0.50.

**Table 3:** Multivariate analysis of variance (MANOVA) factorial (2^a^ × 2^b^ × 2^c^) for parental practices and dimensions.

Source of Variation	Λ	*F*	*df*_1_	*df*_2_	*p*	*η* ^2^
(A) Sex ^a^	0.932	5.76	9	710	<0.001	0.068
(B) Age ^b^	0.874	11.38	9	710	<0.001	0.126
(C) Country ^c^	0.673	38.39	9	710	<0.001	0.327
A × B	0.990	0.77	9	710	0.639	0.010
A × C	0.995	3.69	9	710	<0.001	0.045
B × C	0.980	1.60	9	710	0.110	0.020
A × B × C	0.988	0.95	9	710	0.484	0.012

Note: ^a^: a1 = female, a2 = male; ^b^: b1 = 12–15 years old, b2 = 16–19 years old; ^c^: c1 = Germany, c2 = Spain; Λ = Wilks’ Λ; *F* = *F* value; *df*_1_ = degrees of freedom for the effect; *df*_2_ = degrees of freedom for the error; *p* = *p*-values; and *η*^2^ = effect size.

**Table 4:** Means and (standard deviations) for gender, age, and country and main univariate *F* values for parental practices and dimensions.

	Sex			Age			Country		
	Female	Male	F(1, 718)	12–15	16–19	*F*(1, 718)	Germany	Spain	*F*(1, 718)
Warmth	3.03 (0.02)	2.96 (0.03)	4.16 *	3.09 (0.03)	2.90 (0.02)	27.31 ***	3.05 (0.02)	2.94 (0.03)	9.76 **
Affection	2.74 (0.04)	2.57 (0.04)	9.33 **	2.81 (0.04)	2.49 (0.04)	33.21 ***	2.74 (0.04)	2.56 (0.04)	10.88 **
Dialog	2.65 (0.03)	2.59 (0.04)	1.86	2.66 (0.04)	2.58 (0.03)	2.85	2.50 (0.03)	2.74 (0.04)	25.10 ***
Indifference	1.83 (0.03)	1.86 (0.04)	0.27	1.72 (0.04)	1.96 (0.03)	24.55 ***	1.69 (0.03)	2.00 (0.04)	40.62 ***
Detachment	1.64 (0.02)	1.64 (0.03)	0.1	1.57 (0.03)	1.71 (0.02)	16.22 ***	1.52 (0.02)	1.77 (0.03)	48.34 ***
Strictness	1.55 (0.02)	1.58 (0.02)	1.41	1.65 (0.02)	1.48 (0.02)	43.03 ***	1.72 (0.02)	1.40 (0.02)	150.89 ***
Verbal scolding	2.15 (0.03)	2.11 (0.03)	0.48	2.25 (0.03)	2.01 (0.03)	30.46 ***	2.36 (0.03)	1.90 (0.03)	106.41 ***
Phys.Punishment	1.04 (0.01)	1.13 (0.01)	26.17 ***	1.10 (0.01)	1.07 (0.01)	2.81	1.13 (0.01)	1.04 (0.01)	31.64 ***
Revok.Privileges	1.45 (0.02)	1.49 (0.03)	1.06	1.59 (0.03)	1.35 (0.02)	42.78 ***	1.67 (0.02)	1.27 (0.03)	122.84 ***

Note: Bonferroni-adjusted comparison α = 0.05; * *p* < 0.05, ** *p* < 0.01, *** *p* < 0.001.

**Table 5:** Factorial multivariate analysis of variance (4^a^ × 2^b^ MANOVA) for adolescent adjustment.

Source of Variation	Λ	*F*	*df*_1_	*df*_2_	*p*	*η* ^2^
(A) Parenting ^a^	0.647	15.93	21	2045	<0.001	0.135
(B) Country ^b^	0.792	26.74	7	712	<0.001	0.208
A × B	0.885	4.23	21	2045	<0.001	0.040

Note: ^a^: a1 = indulgent, a2 = authoritative, a3 = authoritarian, a4 = neglectful; ^b^: b1 = Germany, b2 = Spain; Λ = Wilks’ Λ; *F* = *F* value; *df*_1_ = degrees of freedom for the effect; *df*_2_ = degrees of freedom for the error; *p* = *p*-values; *η*^2^ = effect size.

**Table 6:** Means and standard deviations (in parentheses) for each country and for each parenting style, and the main univariate values of *F* for the factors of self-concept, academic performance, and substance use.

	Country			Parenting Style				
	Germany	Spain	*F*(1, 718)	Indulgent	Authoritative	Authoritarian	Neglectful	*F*(3, 718)
Academic SC	6.02 (0.09)	6.79 (0.10)	35.06 ***	7.24 (0.14) ^1^	6.47 (0.12) ^2a^	6.12 (0.14) ^2^	5.79 (0.12) ^2b^	22.33 ***
Social SC	7.06 (0.08)	7.52 (0.09)	15.19 ***	7.59 (0.13) ^1^	7.43 (0.11) ^1a^	6.98 (0.13) ^2b^	7.15 (0.11) ^2^	5.00 **
Emotional SC	5.27 (0.10)	6.78 (0.10)	114.43 ***	6.17 (0.15)	5.65 (0.13)	6.03 (0.15) ^2^	6.26 (0.13) ^1^	4.17 **
Family SC	7.90 (0.08)	8.28 (0.09)	10.02 **	9.21 (0.13) ^1^	8.36 (0.11) ^2a^	6.65 (0.13) ^2b^	8.13 (0.11) ^2a^	66.79 ***
Physic SC	5.64 (0.09)	6.53 (0.10)	41.66 ***	6.27 (0.15)	6.45 (0.13) ^1^	5.81 (0.15) ^2^	5.82 (0.12) ^2^	5.76 ***
A. Achiev	5.26 (0.07)	5.14 (0.07)	1.64	5.70 (0.10) ^1^	5.59 (0.09) ^1^	4.85 (0.10) ^2^	4.68 (0.09) ^2^	30.12 ***
Substances	1.55 (0.03)	1.45 (0.03)	5.22 *	1.44 (0.05) ^2^	1.36 (0.04) ^2b^	1.63 (0.05) ^1^	1.57 (0.04) ^1a^	7.38 ***

Note: Bonferroni-adjusted comparison α = 0.05; ^1^ > ^2^; ^a^ > ^b^; * *p* < 0.05, ** *p* < 0.01, *** *p* < 0.001.

## Data Availability

The datasets generated and/or analyzed during the current study are available from the corresponding author upon reasonable request.
